# Intra-observer and interobserver variability of biventricular function, volumes and mass in patients with congenital heart disease measured by CMR imaging

**DOI:** 10.1186/1532-429X-11-S1-P100

**Published:** 2009-01-28

**Authors:** Saskia E Luijnenburg, Daniëlle Robbers-Visser, Adriaan Moelker, Hubert W Vliegen, Barbara JM Mulder, Willem A Helbing

**Affiliations:** 1 Erasmus MC, Rotterdam, Netherlands; 2 Leiden University Medical Center, Leiden, Netherlands; 3 Amsterdam Medical Center, Amsterdam, Netherlands

**Keywords:** Left Ventricular Ejection Fraction, Right Ventricular, Congenital Heart Disease, Cardiovascular Magnetic Resonance, Interobserver Variability

## Objective

The aim of this study was to assess intra-observer and interobserver variability of biventricular function, volumes and mass in a heterogeneous group of patients with congenital heart disease (CHD) using cardiovascular magnetic resonance (CMR) imaging.

## Background

CMR imaging provides highly accurate measurements of biventricular volumes and mass and is frequently used in the follow-up of patients with acquired and congenital heart disease. Data on reproducibility are limited in patients with CHD, while measurements should be reproducible, since CMR imaging has a main contribution to decision making and timing of (re)interventions.

## Methods

Thirty-five patients with CHD (26 males, 9 females; age range 7 – 62 years) were included in this study. A short axis set of contiguous slices was acquired with CMR imaging using a steady-state free precession (SSFP) pulse sequence. Intra-observer and interobserver variability was assessed for left ventricular (LV) and right ventricular (RV) volumes, function and mass by calculating the coefficient of variability.

## Results

Intra-observer variability was between 2.9% and 6.8%, with the smallest variations in LV and RV end-diastolic volume (EDV) and the highest variations in LV end-systolic volume (ESV) and RV mass. Interobserver variability was between 3.9% and 10.2%, with the smallest variations in LV ejection fraction (EF) and RV EDV and the highest variations in biventricular ESV and mass.

## Conclusion

Intra-observer and interobserver variability of biventricular parameters assessed by CMR imaging is good for a heterogeneous group of patients with CHD. CMR imaging is an accurate and reliable method for follow-up of biventricular function, volumes and mass and should allow adequate assessment of changes in ventricular size and global ventricular function.

Supported by NHF grant 2006B095

